# Preliminary Study of MR Diffusion Tensor Imaging of Pancreas for the Diagnosis of Acute Pancreatitis

**DOI:** 10.1371/journal.pone.0160115

**Published:** 2016-09-01

**Authors:** Xinghui Li, Ling Zhuang, Xiaoming Zhang, Jian Wang, Tianwu Chen, Liangjun Li, Emmanuel Ajedichiga Aduah, Jiani Hu

**Affiliations:** 1 Department of Radiology, The First Affiliated Hospital of the First Military Medical University, Chongqing, China; Department of Radiology, Affiliated Hospital of North Sichuan Medical College, Nanchong, China; 2 Department of Radiation Oncology, Wayne State University, Detroit, Michigan, United States of America; 3 Department of Surgery, Affiliated Hospital of North Sichuan Medical College, Nanchong, China; 4 Department of Radiology, Wayne State University, Detroit, Michigan, United States of America; Shenzhen institutes of advanced technology, CHINA

## Abstract

**Objectives:**

To evaluate the feasibility of differentiating between acute pancreatitis (AP) and healthy pancreas using diffusion tensor imaging (DTI) and correlate apparent diffusion coefficient (ADC) /fractional anisotropy (FA) values with the severity of AP.

**Material and Methods:**

66 patients diagnosed with AP and 20 normal controls (NC) underwent DTI sequences and routine pancreatic MR sequences on a 3.0T MRI scanner. Average ADC and FA values of the pancreatic were measured. Differences of FA and ADC values between the AP group and the NC group with AP and healthy pancreas were compared by two-sample independent t-test. The severity of AP on MRI was classified into subgroups using MR severity index (MRSI), where the mean FA and ADC values were calculated. Relationship among the FA values, ADC values and MRSI were analyzed using Spearman's rank correlation coefficients.

**Results:**

The pancreatic mean ADC value in the AP group (1.68 ± 0.45×10^−3^mm^2^/s) was significantly lower than in the NC group (2.09 ± 0.55×10^−3^mm^2^/s) (P = 0.02); the same as mean FA value (0.39 ± 0.23 vs 0.54 ± 0.12, P = 0.00). In the subgroup analysis, the pancreatic ADC and FA value of edema AP patients was significantly higher than necrosis AP patients with P = 0.000 and P = 0.001respectively. In addition, as severity of pancreatitis increased according to MRSI, lower pancreatic ADC (r = -0.635) and FA value (r = -0.654) were noted.

**Conclusion:**

Both FA and ADC value from DTI can be used to differentiate AP patients from NC. Both ADC and FA value of pancreas have a negative correlation with the severity of AP.

## Introduction

Acute pancreatitis (AP) is a non-bacterial inflammatory disease of the pancreas characterized by auto-digestion of the pancreatic parenchyma and peripancreatic tissues. The incidence of AP is increasing worldwide with approximately 210,000 people admitted to the hospital in the United States annually of which about 5% die [1, 2]. Among all the AP patients, 20–30% of AP patients develop severe necrosis pancreatitis with high morbidity and mortality rate up to 20% -45% [3, 4]. Therefore, it is critical to differentiate AP from healthy pancreas and grade AP severity in clinical practice.

Several studies have recently applied DWI in evaluating acute and chronic pancreatic inflammation [5–9]. Diffusion MRI, particularly DWI, is increasingly used in routine abdominal MRI protocols [5], while the apparent diffusion coefficient (ADC) calculated from DWI has been used to reveal quantitative molecular diffusion. However, DWI merely provides an average ADC over three orthogonal directions, disregarding the anisotropy of tissue structure [10, 11]. It is well known that pancreas is an anisotropic endocrine organ with plenty of vascular supply, as well as complicated anatomy and physiological features [12–14]. Therefore, DWI and its ADC map may not provide accurate anisotropy diffusion characteristics of extracellular water molecules of AP [5].

Diffusion Tensor Imaging (DTI), on the other hand, is a noninvasive diffusion MRI which can provide anisotropy water diffusion information by calculating the diffusion tensor in at least six gradient directions for every section in DTI gradients [15]. Quantitative index of fractional anisotropy (FA) value obtained by DTI is used to characterize the degree of diffusion anisotropy [16]. Moreover, through providing additional information on anisotropy diffusion to total diffusion orientations, DTI can provide more precise ADC calculation [17]. Therefore, DTI holds great capability to detect the changes of degree of diffusion anisotropy and molecular diffusion in AP patients by calculating the FA and ADC values of the pancreas. Nevertheless, the feasibility of DTI to distinguish between AP and healthy pancreas has not been investigated yet.

A number of scoring systems are available for assessing the severity of AP, which mainly falls in two categories: clinical evaluation methods including Ranson’s score and APACHE II score; radiologic evaluation methods including CT severity index (CTSI) and MR severity index (MRSI) [18–20]. The Ranson’s score, regrettably is routinely calculated days apart from the clinical course [18]. Compared with Ranson’s score, the APACHE II score can be calculated within hours of hospital admission and is more accurate in the prediction of severity in AP. However, Tang, W et al [18] claimed that MRSI is superior to APACHE II in assessing local complications from pancreatitis. Among all the methods, CTSI based on combined assessment of peripancreatic fluid collections and the degree of pancreatic necrosis, was considered as the golden standard to assess the degree of severity, extent of necrosis, local complications and prognosis of clinical outcome [21]. Nevertheless, CT involves radiation exposure and iodinated contrast media, which is not suitable for patients with functional renal impairment or a history of allergic-type reactions to iodinated contrast material[1]. Arvanitakis M et al [22] and Stimac D et al [23] demonstrated that MRSI had a significant correlation with CTSI on C-reactive protein (CRP) levels 48 hours after admission, clinical outcome, length of hospital stay. Therefore, MRSI is chosen as the standard to stage AP severity in this study.

DTI has been used predominantly for brain imaging to characterize the healthy and diseased tissues in brain white matter [24–27]. Nissan N et al [10] has applied DTI in identifying significant changes in the DTI measurements of pancreatic ductal adenocarcinoma as compared to normal pancreatic tissue. They established that DTI could characterize of the water diffusion and anisotropy of the healthy pancreas. However, a clinical evaluation exploring the feasibility of DTI for differentiating between AP and healthy pancreas has not yet been reported, as well as the relationship between FA/ADC values and the severity of AP. Therefore, we conducted this study to measure and compare FA and ADC values in patients with AP to healthy pancreas using DTI, as well as evaluating the correlations between FA, ADC values and the severity of AP according to MRSI.

## Materials and Methods

### Patient population

#### AP patients and normal controls

78 consecutive patients with a clinical history of AP and 20 normal controls in our institute between October 2013 and July 2014, and March 1st to April 25th in 2016 were initially considered in this study. The 20 normal controls are all volunteers who were in good health without any significant medical history, including diabetes or any other pancreatic diseases, etc. Healthy volunteers with physical examinations, their history, imaging results, laboratory tests were used as diagnostic criteria. The patients are selected upon the following criteria: (1) acute history; (2) first onset of pancreatitis; (3) 3-times-elevated amylase or lipase, with other causes of elevated enzymes excluded; (4) a maximum three-day interval between the MRI examination and the pancreatitis onset.

The criteria for exclusion were the following: (1) chronic pancreatitis; (2) intra- or retroperitoneal tumors, inflammation or hemorrhagic diseases; (3) poor compliance in MR examination and (4) hypoproteinemia [1]. 12 patients met the exclusion criteria (3 met Condition 1, 2 met Condition 2, 5 met Condition 3, 2 met Condition 4) and were excluded from the final study.

The final study group consisted of 66 consecutive patients (29 females; age range 27–75 years, mean age 41.3 ± 34.7years) and 20 normal controls (8 females, age range27–62 years, mean age 27.5 ± 4.6 years).

#### MR imaging technique

All subjects were scanned in the supine position on a 3.0 T MR scanner (Discovery MR 750; GE Medical Systems, Milwaukee, WI.) with a 50 mT/m maximum gradient length and 200 T/m/s maximum slew rate using a 32-channel body array coil with sixteen anterior and sixteen posterior elements. Each patient underwent routine pancreatic MR scans shown in Table 1.

**Table 1:** The parameters of routine pancreatic MR sequences at 3.0T.

	TR	TE	Flip angle	Thickness	Gap	Matrix	Fov
AX 3D LAVA-Flex	4.2	2.6/1.3	15–20°	5	0	384×224	26–33
AX FRFSE T2WI	10000–12000	90–100	90°	5	0.5	256×192	36×34
COR SSFSE T2WI	2500–3500	80–100	90°	5	0.5	384×256	39×33
AX SSFSE T2WI	2500–3500	80–100	90°	5	0.5	320×256	39×33
MRCP	3045	1300	90°	40	40–50	384×224	32×34
AX 3D LAVA C+*	4.2	2.6/1.3	15–20°	5	0	384×224	26–33

**Note:** TR/TE: ms; thickness: mm; gap: mm; fov: cm. Dynamic enhanced imaging is indicated with *. Gadolinium chelate (Magnevist, Schering Guangzhou Co, China) was administered intravenously (0.2 mmol/L per kilogram of body weight) at approximately 3.5 mL/s using a double tube high-pressure injector (Spectris MR Injection System, Medrad Inc, USA) and was followed by a 20 mL saline solution flushed at the same speed. Affter the beginning of the injection, two arterial phase images were created in 19 seconds; two portal vein phase images in 60 seconds and one equilibrium phase image in 180 seconds were obtained.

Thirty minutes before MR examination in each patient, respiratory training should be prepared well. We repeatedly trained patients to hold breath more than 22 seconds using independent ways or families with assistance. Patients with poor compliance in breath-holding were excluded from the study.

After a gradient echo localizer, DTI was acquired in axial orientation with a breath-hold fat saturated single spin echo planar imaging before contrast agent injected. The parameters were as following: TR 2500 ms; TE minimum ms; section thickness = 5 mm; intersection gap = 0 mm; FOV = 28–34 cm, bandwidth 250 Hz; NEX = 1; and matrix = 256×192; Auto shim: on.

Considering the NED and the choice of b-values are two key factors in DTI data acquisition, which can affect imaging quality, scan time and FA/ADC values, the choice of NED and b-values pancreas DTI is vital in clinical practice. Therefore, before starting this experiment, an optimal set of DTI parameters was obtained from a group of fifteen volunteers with multiple b-values (0,100), (0, 300), (0, 500) and (0, 800) s/mm^2^ and various diffusion-encoding directions (NED = 6, 9, and 12). The DTI acquisitions in each volunteer were repeated 12 times with multiple number of encoding directions of 6, 9, 12 and b = values of 100, 300, 500, and 800 s/mm^2^ respectively. After the statistical analyzing of the quantitative and qualitative imaging quality, as well as FA/ADC values, an optimal set of DTI parameters with NED = 9 and b-value = (0, 500) s/mm^2^ was selected to achieve a breath-held pancreas DTI scan in 22 seconds on a 3T MRI scanner (GE Discovery MR 750). Details of the optimization can be found in another submission of us[28], which is not the focus of this study.

In total, 86 subjects were enrolled in this study, they included 66 AP patients and 20 normal pancreas controls. Each one was scanned successfully and no morphologic abnormalities were found.

### MR image analysis

#### Grading the severity of AP

The grade of pancreatitis was evaluated independently by two radiologists (with 6 and 7 years of experience in abdominal MR images respectively) based on the 46 patients’ MRI data using the Advantage Workstation 4.4 (GE Healthcare). AP was determined as edematous and necrotic pancreatitis based on MR images. Pancreatic necrosis was defined as a well-marginated area of signal intensity(SI) different from the SI of a normal pancreas on non-enhanced imaging, as well as the absence of enhancement on contrast imaging[29]. Acute edematous pancreatitis was defined as no pancreatic necrosis. The severity of AP was graded according to the MR severity index (MRSI) classified as mild (0–3 points), moderate (4–6 points), or severe (7–10 points)[12] (Table 2). The inter-observer agreement at the presence of scores was obtained for each image, and consensus was reached on cases that were graded differently between the two readers and used in the final analysis.

**Table 2:** MRSI scoring system.

Prognostic indicators	Characteristics	Point
Inflammation	Normal pancreas	0
	Focal or diffuse enlargement of the pancreas	1
	Intrinsic pancreatic abnormalities with inflammatory changes in the peripancreatic fat	2
	Single, poorly defined fluid collection or phlegmon	3
	Two or more poorly defined collection or presence of gas in or adjacent to the pancreas	4
Necrosis	No necrosis	0
	<30%	2
	30–50%	4
	>50%	6

#### DTI Indices

An average diffusion coefficient along each direction was derived from the DW images, as follows:

$$S\left(i\right)=S_{0}\times e^{-b_{i}\times ADC}$$

[30]

Where S(i) is the signal intensity measured on the ithb-value image and bi is the corresponding b-value. S_0_ is the exact signal intensity for a b-value = 0 s/mm^2^.

The eigenvectors eigenvalues (λ1, λ2, λ3) of the diffusion tensor were determined. The primary eigenvalue λ1 (axial or longitudinal diffusivity) was the largest and least restricted diffusivity and the secondary and tertiary eigenvalues (λ2, λ3) (and their average) reflect lower restricted diffusion orthogonal to V1 (radial or transverse diffusion)[[31]]. The index related to diffusional anisotropy was FA,

$$FA=\frac{\sqrt{3}}{\sqrt{2}}\frac{\sqrt{{\left(\lambda_{1}-\bar{\lambda }\right)}^{2}+{\left(\lambda_{2}-\bar{\lambda }\right)}^{2}+{\left(\lambda_{3}-\bar{\lambda }\right)}^{2}}}{\sqrt{\lambda_{1}^{2}+\lambda_{2}^{2}+\lambda_{3}^{2}}}$$

With $\bar{\lambda }$ being defined as

$$\bar{\lambda }=\frac{\lambda_{1}+\lambda_{2}+\lambda_{3}}{3}$$

[32].

The range of FA value is from 0 to 1. Structures allowing diffusion is absolutely limited and only along a single direction, a maximum FA value of 1 is expected, whereas structures allowing completely free or isotropically restricted diffusion should result in a FA of 0.

#### DTI measurements between AP patients and normal control

**Fig 1:**
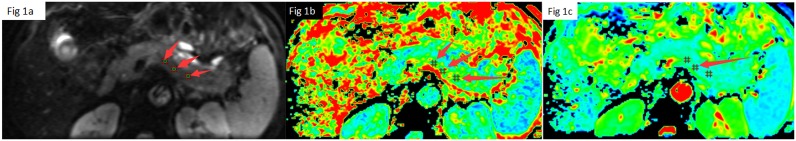
Three typical same ROIs placement of pancreatic body of healthy group on signal intensity image (a), ADC map (b) and FA map (C).

**Fig 2:**
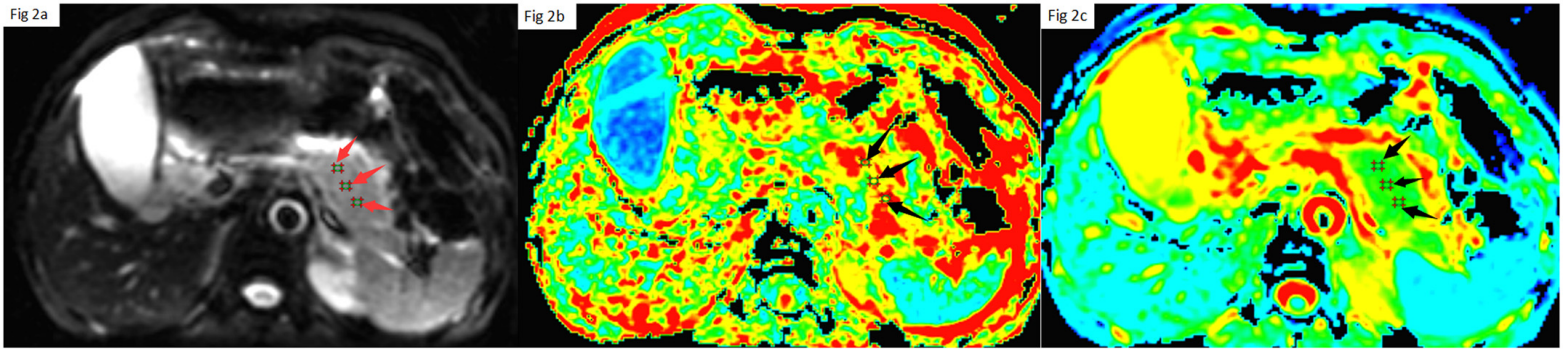
Three typical same ROIs placement within the highest SI area in pancreas in AP group on signal intensity image (a), ADC map (b) and FA map (C).

The original MRI data were loaded onto a workstation (Advantage Workstation 4.4; GE Healthcare). The FuncTool 2 software was used to process all DTI raw data and then the ADC maps and FA maps were obtained automatically. We removed the surrounding fat, bone, gas and other tissue image applying the threshold definition method. One radiologist with 10 years of experience in abdominal pancreatic MR images drew the pancreatic regions of interest (ROIs). Pancreatic ROIs were manually delineated on the b = 0 images with the aid of the T2-weighted images, and then transferred automatically to the parametric maps where DTI parameters (FA and ADC values) were calculated. In the normal control group, we calculated the mean FA and ADC values of pancreatic parenchyma by placing three 20 mm^2^ sized circular ROIs over pancreatic body segment (Fig 1). In the AP group, the FA and ADC values from three circular ROIs within the highest SI area in pancreas were measured[6] (Fig 2). Areas of with artifacts pancreatic duct, cystic lesions, and pseudocysts were excluded from the ROI.

#### Statistical analysis

To compare the differences of FA and ADC values between AP and normal controls, two-sample independent t-test were used. The FA and ADC values of the AP patients in each subgroup (included edematous and necrotic AP and mild, moderate, and severe AP according to MRSI) were compared with each other as well as correlations between FA, ADC values and the severity of AP according to (MRSI) using the two-sample independent t-test and One-way ANOVA: Post Hoc Multiple Comparison- Bonferroni. The relationship between the FA values and the severity of AP based on MRSI, was analyzed using Spearman's rank correlation coefficients respectively, the same as between the ADC values and MRSI. All statistical analyses were performed using Statistical Package for Social Sciences (SPSS) for Windows (Version 13.0, Chicago, IL, USA). P values≤0.05 were considered indicative of a statistically significant difference.

#### Ethics Statement

The study was approved by the ethics committee of Affiliated Hospital of North Sichuan Medical College. Healthy volunteers were asked to voluntarily participate in this study, there was no specific protocol or methodology on the selection of the participants of this study as this was a convenience sample. However, a verbal informed consent regarding the goals of the study and the willingness to participate was taken by the AP participants. Before the ethical approval, the proposal was provided to reviewers to assure the ethical issues. Finally, the ethical review committee approved the oral consent by considering the study qualified as involving only “minimal risks" to participants. Before MRI examination, the interviewer fully explained the purpose of the study to each participant and obtained full verbal informed consent from each study participant.

## Results

### Medical history of AP patients

In the 66 patients with AP, the etiology of AP was biliary in 50.0% (33/66), alcoholic in 7.6% (5/66), and traumatic in 1.5% (1/66). Forty-one percent (27/66) of the patients did not have a specified etiology. 28.7% (19/66) underwent gallstone surgery, 45.4% (30/66) patients suffered from fatty liver disease, 7.58% (5/66) patients had hypertension, 30.3% (20/66) patients were with liver cysts and 25.8% (17/66) patients with renal cysts, 9.1%(6/66) patients had a history of chronic gastritis.

#### Grading the severity of AP

**Fig 3:**
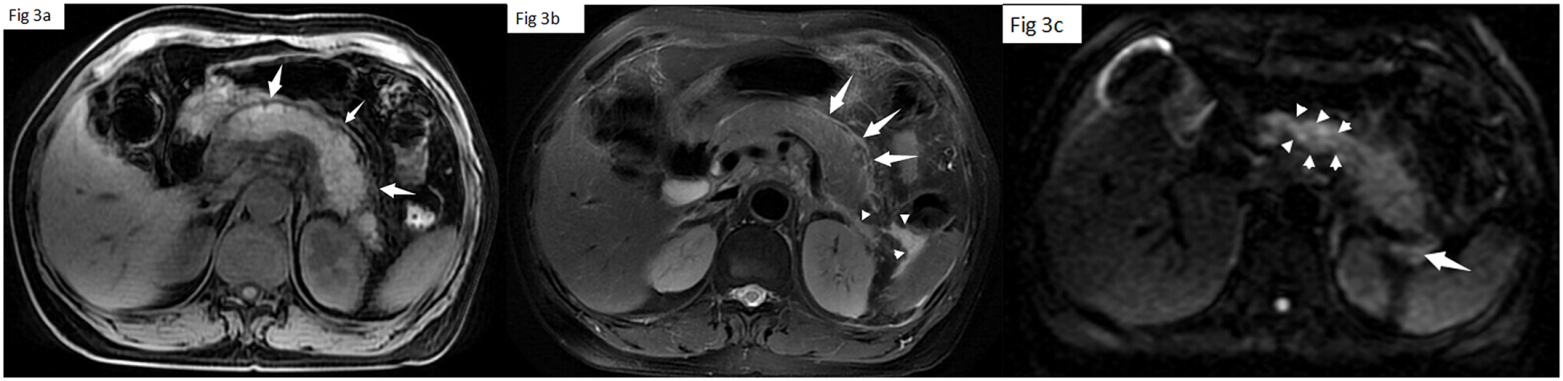
A 40-year-old woman with mild AP. Fat suppression Lava-flex T1 (a) and FRFSE T2 (b) weighted images show the swollen pancreatic tissue, the thickened fascia of pancreas (arrows) and edematous peripancreatic fat (arrowhead). DTI(c) shows the increased signal in pancreatic parenchyma, particularly pancreatic head (arrowhead) and fuzzy peripancreatic fat (arrow).

**Fig 4:**
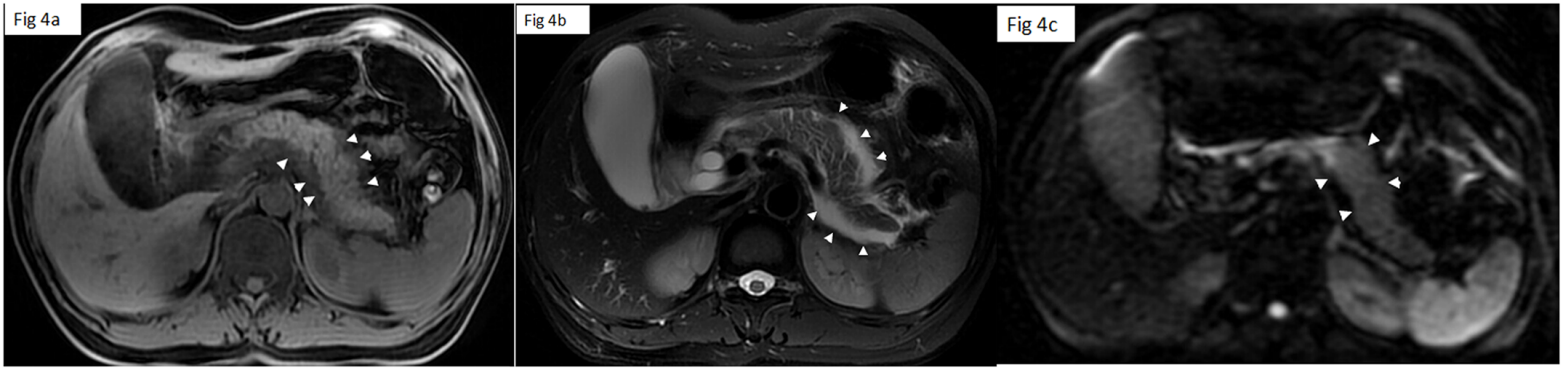
A 43-year-old woman with moderate AP and cholecystolithiasis. Fat suppression Lava-flex T1 (a) and FRFSE T2 (b) weighted images show the swollen pancreatic tissue, and edematous peripancreatic fat (arrowhead). DTI(c) shows the swollen pancreatic parenchyma.

**Fig 5:**
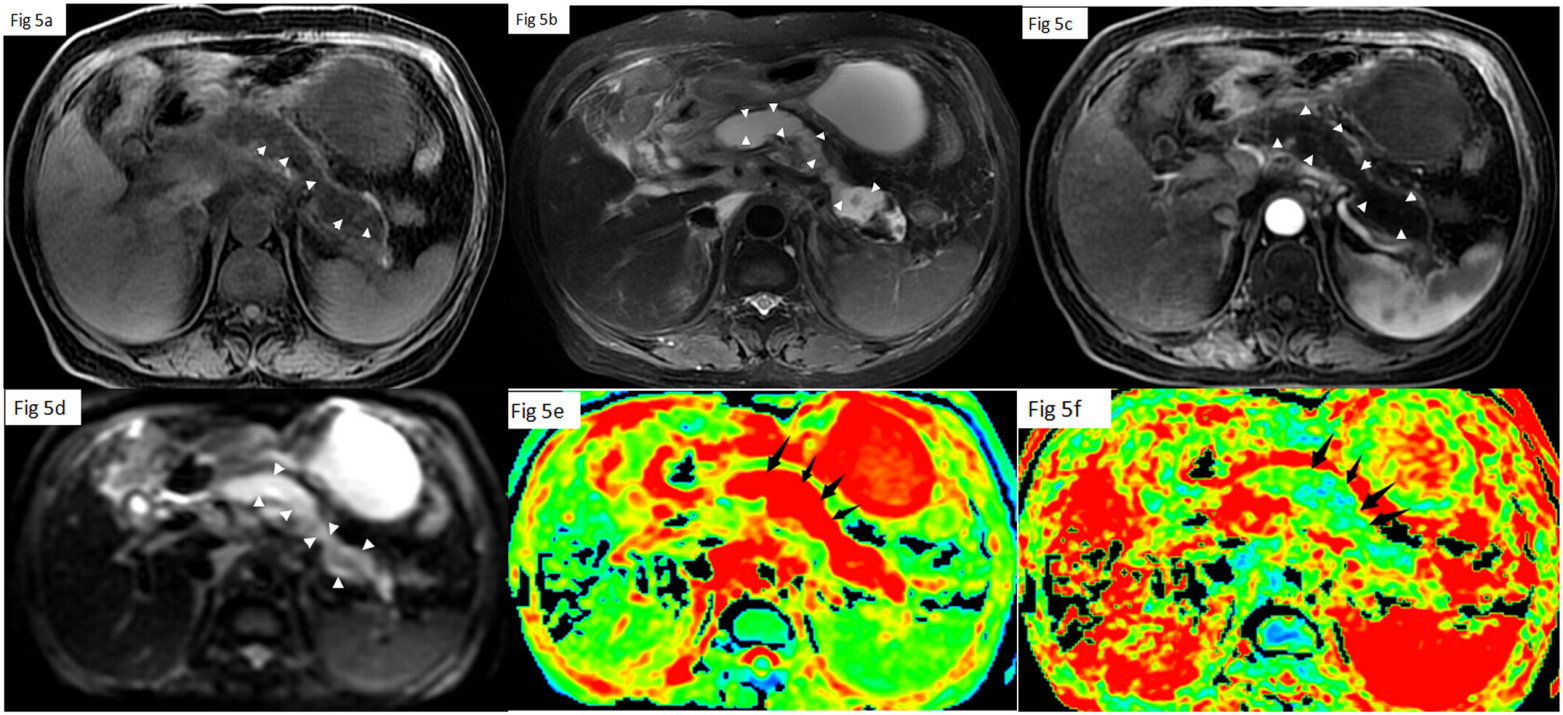
A 76-year-old woman with severe necrosis AP. Lava-flex T1 (a), FRFSE T2 (b), contrast Lava-flex T1(c), DTI(d), ADC map (e) and FA map (f) weighted images show a well-marginated necrosis area without enhancement located in the whole pancreatic tissue(arrow) and a pseudocyst in omental sac.

On MRI images, 84.8% (56/66) patients were diagnosed with edematous AP, while 15.1% (10/66) patients were diagnosed with necrotizing AP. Based on the MRSI, 36.4% (24/66) of the patients had mild AP(Fig 3), 50.5%(33/66) had moderate AP(Fig 4) and 13.6% (9/66) had severe AP(Fig 5).

#### DTI measurements between AP patients and normal control

**Fig 6:**
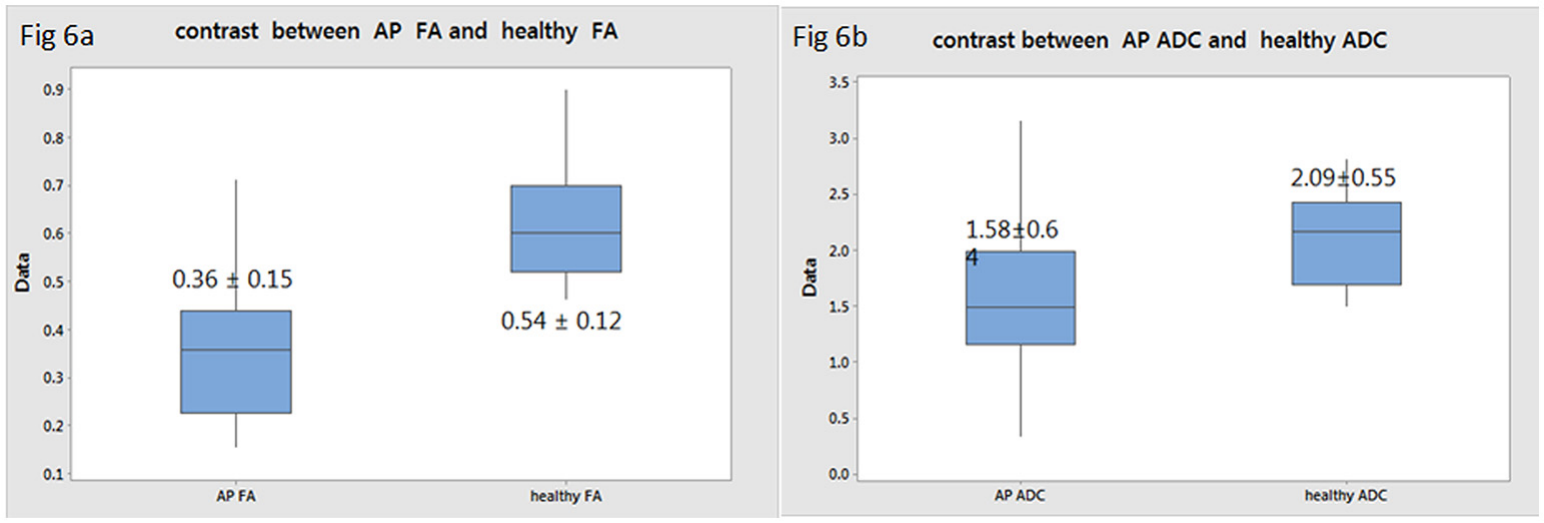
The pancreatic mean ADC value (a) and FA value (b) contrast between in the AP group and normal group, we can gain that the pancreatic ADC value and FA value in the AP group was significantly lower than in the normal group.

The pancreatic mean ADC value in the AP group (1.68±0.45×10^−3^mm^2^/s) was significantly lower than in the normal control group (2.09±0.55×10^−3^mm^2^/s) (P = 0.02); the same as mean FA value (0.39 ± 0.23 vs 0.54 ± 0.12, P = 0.00) (Fig 6). The pancreatic FA and ADC values in the edematous AP subgroup were both higher than that of the necrotizing AP subgroup (P = 0.00 /0.00) (Table 3). The ADC values of pancreas in mild, moderate, and severe AP (based on the MRSI) were 2.03 ± 0.19×10^−3^mm^2^/s, 1.78 ± 0.15×10^−3^mm^2^/s, 1.34 ± 0.49×10^−3^mm^2^/s, respectively, and the FA values were respectively 0.54 ± 0.20, 0.49 ± 0.19, 0.16 ± 0.05 based on MRSI. The pancreatic FA and ADC values was correlated with the AP severity as determined by the MRSI (r = -0.63, -0.65; P < 0.01) (Table 4).

**Table 3:** Comparison of the FA and ADC value between edematous and necrotic AP.

Parameter	Edematous AP	Necrotic AP	*P* Value
ADC value	1.95 (0.44)	1.39 (0.18)	0.000*
FA value	0.55(0.17)	0.18 (0.06)	0.001*

Note: The data are the mean ADC and FA ((standard deviation). The ADC values were equal to mean value×10−3mm^2^/s. Significant differences (P<0.05) are indicated with *.

**Table 4:** Correlation of the pancreas ADC and FA value with AP severity determined by the MRSI.

ADC value (×10^−^3mm^2^/s)	AP subgroups based on MRSI			*R* value	*P* Value
	Mild	Moderate	Severe		
ADC value	2.03 (0.52)	1.78 (0.19)	1.34(0.15)	**-0.635**	0.003*
FA value	0.54(0.20)	0.49 (0.19)	0.16(0.04)	**-0.654**	0.002*

Note: The data are the mean ADC and FA ((standard deviation). Significant differences (P<0.05) are indicated with *.

## Discussion

Our study demonstrated the feasibility of differentiating AP and healthy pancreas using DTI, as well as investigated the role of FA/ADC values as indicators to predict the severity of AP.

FA value is used to characterize the degree of diffusion anisotropy[16]. In our study, FA value in patients with AP was observed significantly lower than normal controls (P = 0.00). It reflects that diffusion in AP is more isotropic than healthy controls and FA values of DTI can be potentially used as a diagnostic indicator for AP. It is mainly due to the anatomy structural and physiological features of pancreas, as well as the pathogenesis of AP. The pancreas is a glandular organ with complex exocrine microstructure and endocrine microvascular physiological features and it would have a good diffusion characteristic with anisotropy diffusion of extracellular water molecules[10]. However, AP is a disease characterized by cytoplasmic vacuolization, the death of acinar cells, edema formation, and infiltration of inflammatory cells into the pancreas. Al-Eryani S et al [33] described in their study that the destruction of both cellular structure and cellular connections is an early event in the development of AP. Klingberg T et al [34] claimed FA value of 0 in brain indicates that (1)the diffusion of extracellular water molecules tends to be isotropic, (2) the fiber bundle is damaged or immature, (3) cell membranes, myelin and the consistency of axonal direction is corrupted or incomplete. Obviously, FA value decreased in AP patients is closely related to the damaged pancreas microscopic structural and physiological features, which induce inflammatory cells invade in a disorganized fashion the process of AP.

In our study, pancreatic ADC value of DTI in AP patients were found significantly lower than normal controls (P = 0.02). Lower ADC values imply water mobility is restricted and cells are densely packed. There is no report using DTI to differentiate AP, yet rather a limited number of studies applied DWI to evaluate acute and chronic pancreatic inflammation [5–9]. Yencilek, et al[5] and Thomas, et al [6] reported AP had restricted diffusion that could be differentiated from normal pancreas using ADC values. This result is consistent with the results of our study using ADC values from DTI.

The correlation of FA/ADC values from DTI was investigated with the severity of AP in our study as well. Pancreatic ADC and FA values were found to have a negative correlation with the severity of AP based on MRSI (r = -0.635, -0.654), while pancreatic ADC and FA value of necrosis AP was found significantly lower than edema AP patients (P = 0.000 and 0.001). AP is staged by grading both the degree of pancreatic and peripancreatic fluid and the extent of pancreatic necrosis. Yencilek, et al [5] found the severity of pancreatitis increased according to the Balthazar classification acquired lower ADC values of AP patients. However, owing to MRI has a better ability to predict local complications and disease prognosis, MRSI is a more reliable method for staging AP severity [22]. The pancreatic microvascular change in hemodynamics is one important pathogenesis of AP disease, which is not only as a part of initiating AP, but also as an important factor in the progression of the mild edematous pancreatitis to sever necrotizing pancreatitis[35]. Ischemia of the pancreas play a key role in the transition from pancreatic edema to necrosis [36]. With pancreatic microcirculation ischemia aggravated, pancreatic glandular tissue appears necrosis and liquefaction, which restricts water molecules diffuse movement and reduces anisotropy diffusion of extracellular water molecules. Similarly, Cheung et al[37] found that both ADC and FA of kidney medulla in renal ischemia reperfusion injury were significantly less (p < 0.01) than those of contralateral intact medulla. Therefore, our results showed that ADC and FA values might be a supplementary indicator for determining the severity of AP.

However, the number of patients with severe pancreatitis 13.6%(10/66) may introduce a slightly biased conclusion for our results. More patients data will be included in our future study. Ertrk et al[11] concluded that DTI provides a more precise ADC calculation than DWI providing information of anisotropy, therefore, we did not compare ADC values between DTI and DWI in AP in this study. FA/ADC values were found to be great indicators for AP diagnosis and determining the severity of AP from this study, which exhibits potential value to clinical practice. Our future work will focus on the sensitivity and specificity of FA/ ADC values in AP diagnosis and grading AP severity.

## Conclusion

In conclusion, pancreas DTI provides valuable information in AP diagnosis and AP severity staging. Pancreatic ADC and FA values are significantly lower in patients with AP than normal controls, which have exhibited a negative correlation with the severity of AP according to MRSI.

## Supporting Information

S1 Checklist PLOS ONE Clinical Studies Checklist. (PDF)

S2 Checklist PLOS ONE Clinical Studies Checklist. (PDF)

S1 Fig ROIs placement in healthy group. (PDF)

S2 Fig ROIs placement in AP group. (PDF)

S3 Fig Images with mild AP. (PDF)

S4 Fig Images with moderate AP. (PDF)

S5 Fig Images with severe necrosis AP. (PDF)

S6 Fig Compare ADC and FA value between the AP group and normal group. (PDF)

S1 Table The parameters of routine pancreatic MR sequences at 3.0T. (PDF)

S2 Table MRSI scoring system. (PDF)

S3 Table Comparison of the FA and ADC value between edematous and necrotic AP. (PDF)

S4 Table Correlation of the pancreas ADC and FA value with AP severity determined by the MRSI. (PDF)
