# Correction: Preliminary Study of MR Diffusion Tensor Imaging of Pancreas for the Diagnosis of Acute Pancreatitis

**DOI:** 10.1371/journal.pone.0165353

**Published:** 2016-10-19

**Authors:** Xinghui Li, Ling Zhuang, Xiaoming Zhang, Jian Wang, Tianwu Chen, Liangjun Li, Emmanuel Ajedichiga Aduah, Jiani Hu

There is a mistake in affiliation 1 for authors Xinghui Li, Xiaoming Zhang, Jian Wang, Tianwu Chen, and Liangjun Li. Affiliation 1 should be: Department of Radiology, The First Affiliated Hospital of the Third Military Medical University, Chongqing, China.

There is an error in the first sentence of the ‘MR image analysis’ subsection within the Materials and Methods section. The correct sentence is: The grade of pancreatitis was evaluated independently by two radiologists (with 6 and 7 years of experience in abdominal MR images respectively) based on the 66 patients’ MRI data using the Advantage Workstation 4.4.
