# Supplementary material for: Preliminary Study of MR Diffusion Tensor Imaging of Pancreas for the Diagnosis of Acute Pancreatitis
Source: PLoS One. 2016 Sep 1;11(9):e0160115. doi: 10.1371/journal.pone.0160115 (PMC5008639; doi:10.1371/journal.pone.0160115)

S1 Fig: Three typical same ROIs placement of pancreatic body of healthy group on signal intensity image (a), ADC map (b) and FA map (C)

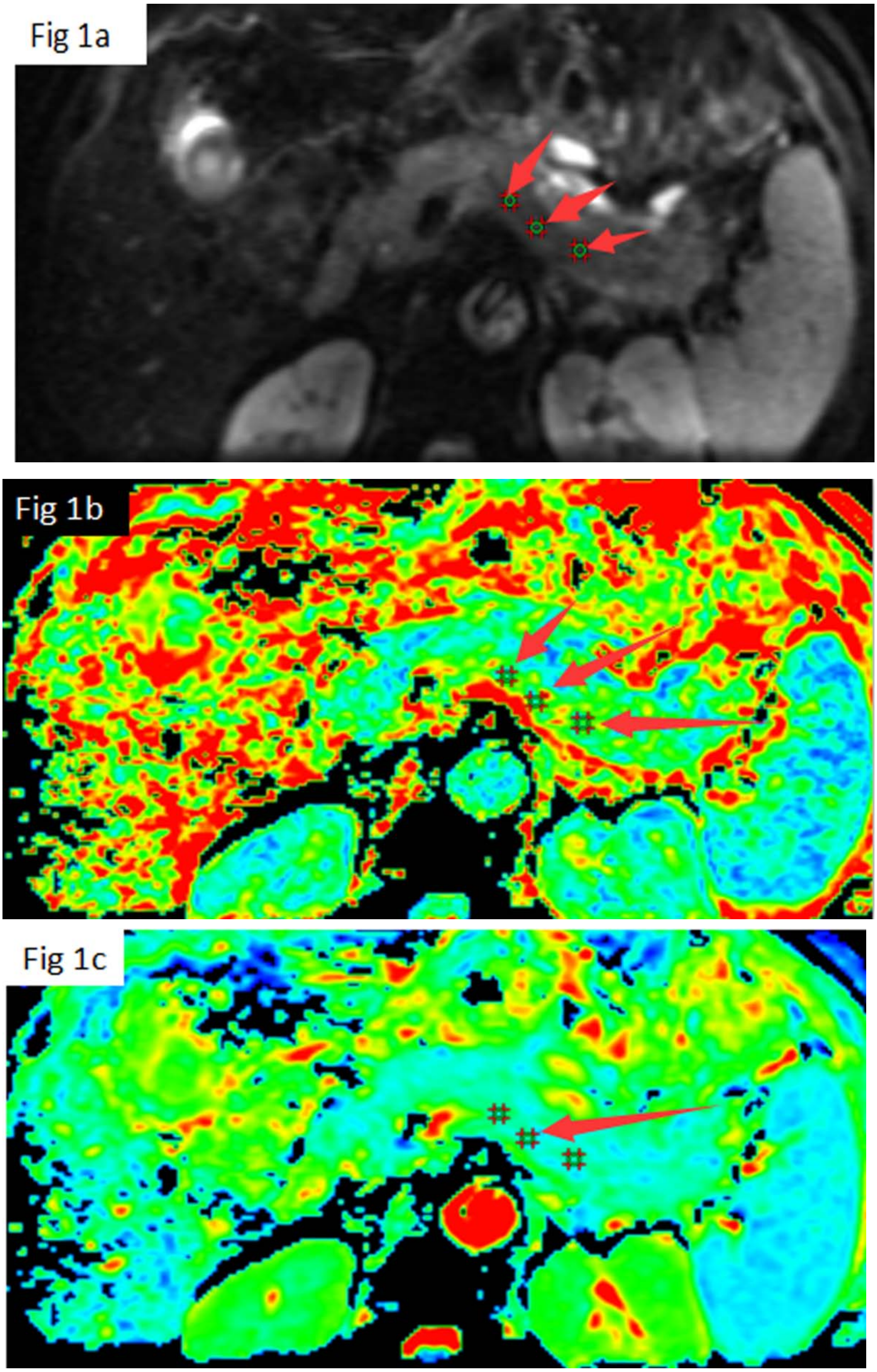

Supplement: S1 Fig — (PDF) [file pone.0160115.s003.pdf]
