# Supplementary material for: Preliminary Study of MR Diffusion Tensor Imaging of Pancreas for the Diagnosis of Acute Pancreatitis
Source: PLoS One. 2016 Sep 1;11(9):e0160115. doi: 10.1371/journal.pone.0160115 (PMC5008639; doi:10.1371/journal.pone.0160115)

S2 Fig: Three typical same ROIs placement within the highest SI area in pancreas in AP group on signal intensity image (a), ADC map (b) and FA map (C).

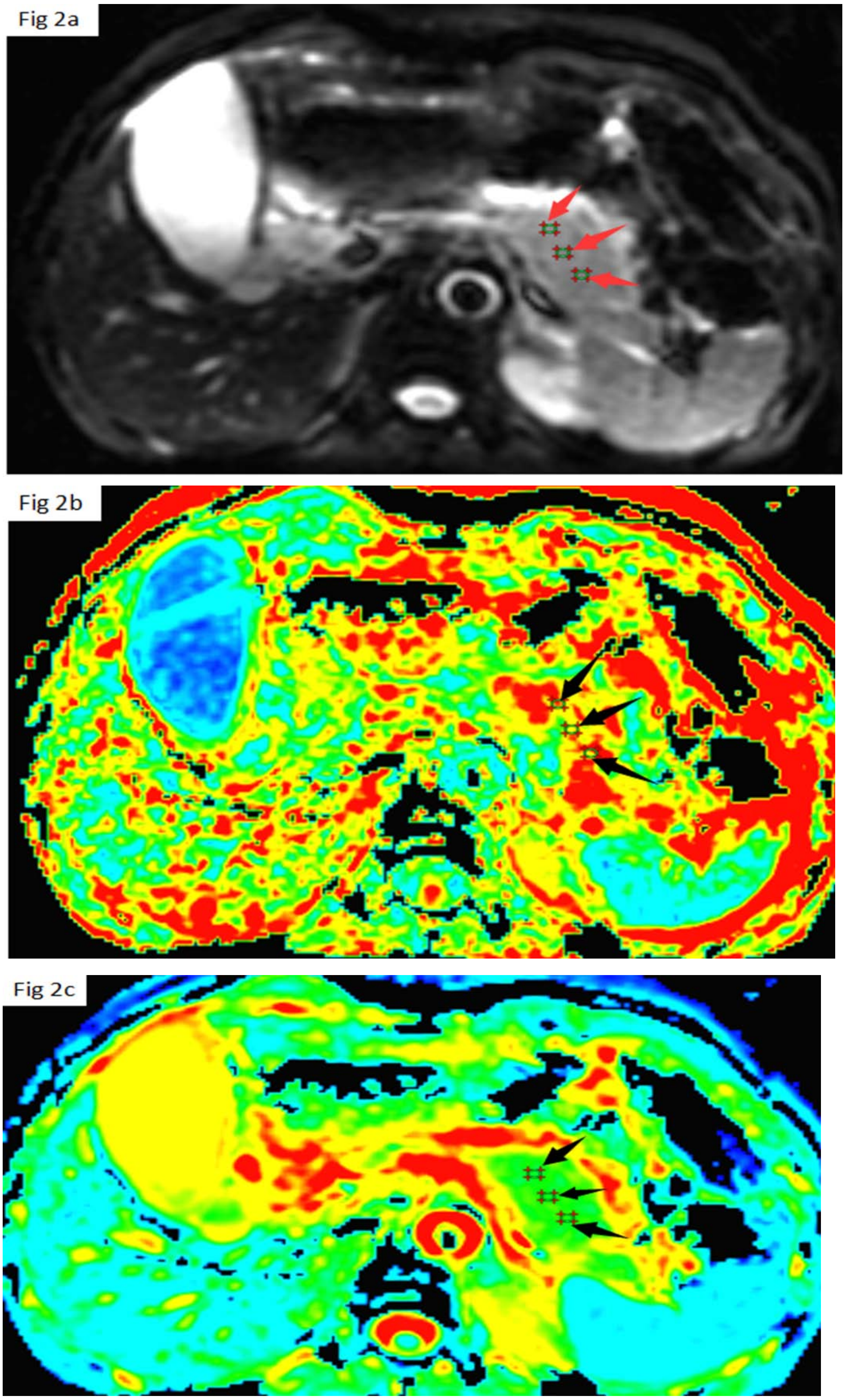

Supplement: S2 Fig — (PDF) [file pone.0160115.s004.pdf]
