# Supplementary material for: Preliminary Study of MR Diffusion Tensor Imaging of Pancreas for the Diagnosis of Acute Pancreatitis
Source: PLoS One. 2016 Sep 1;11(9):e0160115. doi: 10.1371/journal.pone.0160115 (PMC5008639; doi:10.1371/journal.pone.0160115)

S3 Fig: A 40-year-old woman with mild AP. Fat suppression Lava-flex T1 (a) and FRFSE T2 (b) weighted images show the swollen pancreatic tissue, the thickened fascia of pancreas (arrows) and edematous peripancreatic fat (arrowhead). DTI(c) shows the increased signal in pancreatic parenchyma, particularly pancreatic head (arrowhead) and fuzzy peripancreatic fat (arrow).

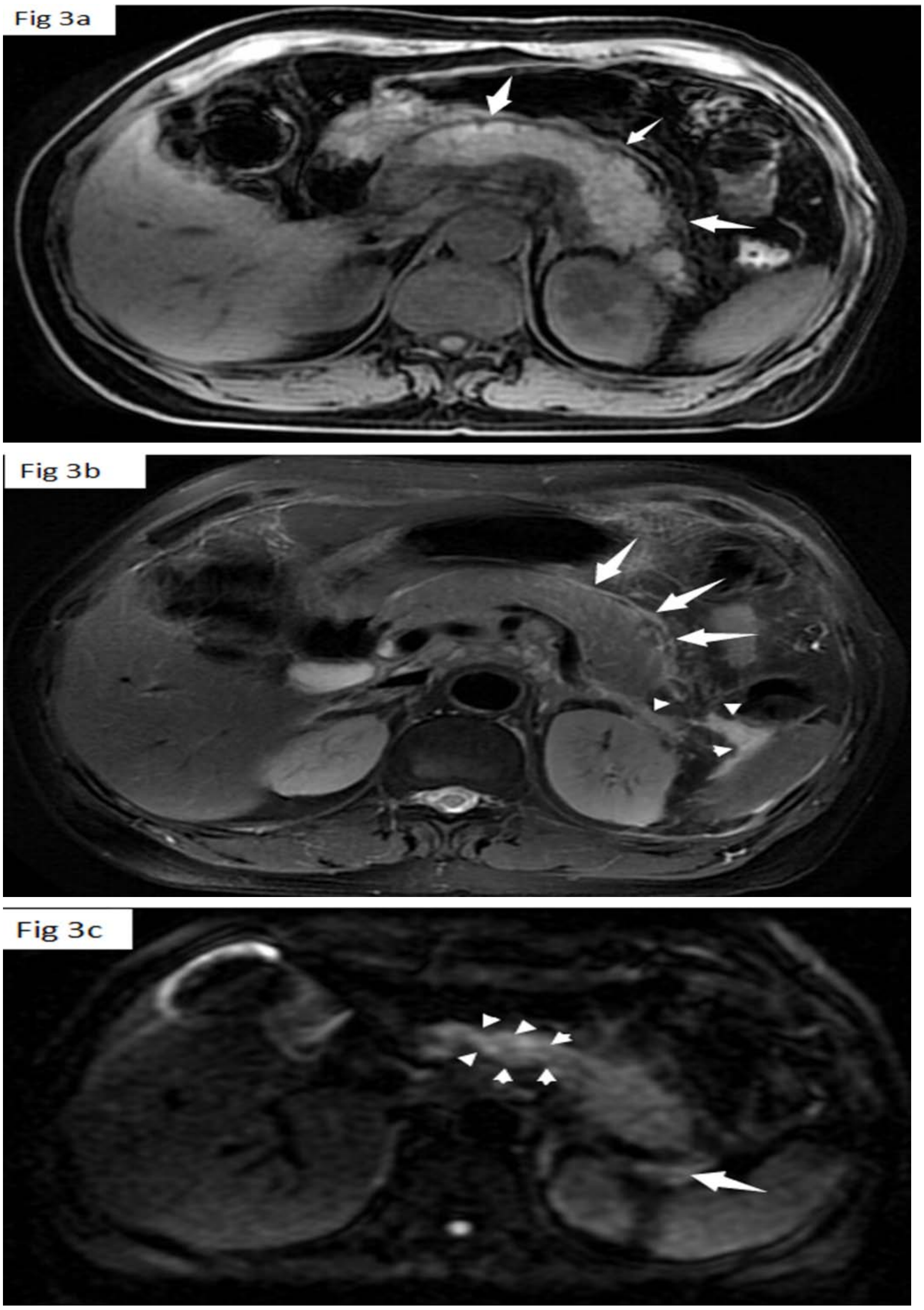

Supplement: S3 Fig — (PDF) [file pone.0160115.s005.pdf]
