# Supplementary material for: Preliminary Study of MR Diffusion Tensor Imaging of Pancreas for the Diagnosis of Acute Pancreatitis
Source: PLoS One. 2016 Sep 1;11(9):e0160115. doi: 10.1371/journal.pone.0160115 (PMC5008639; doi:10.1371/journal.pone.0160115)

S4 Fig: A 43-year-old woman with moderate AP and cholecystolithiasis. Fat suppression Lava-flex T1 (a) and FRFSE T2 (b) weighted images show the swollen pancreatic tissue, and edematous peripancreatic fat (arrowhead). DTI(c) shows the swollen pancreatic parenchyma.

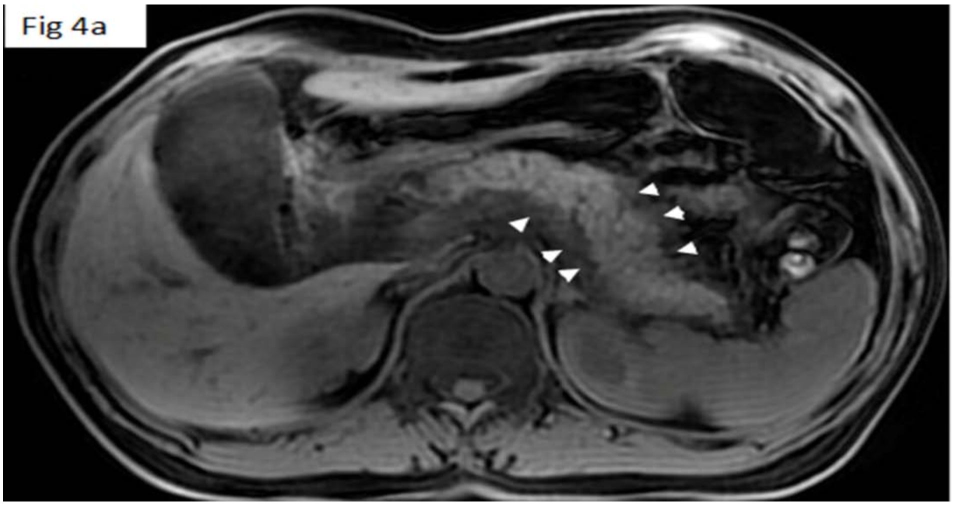

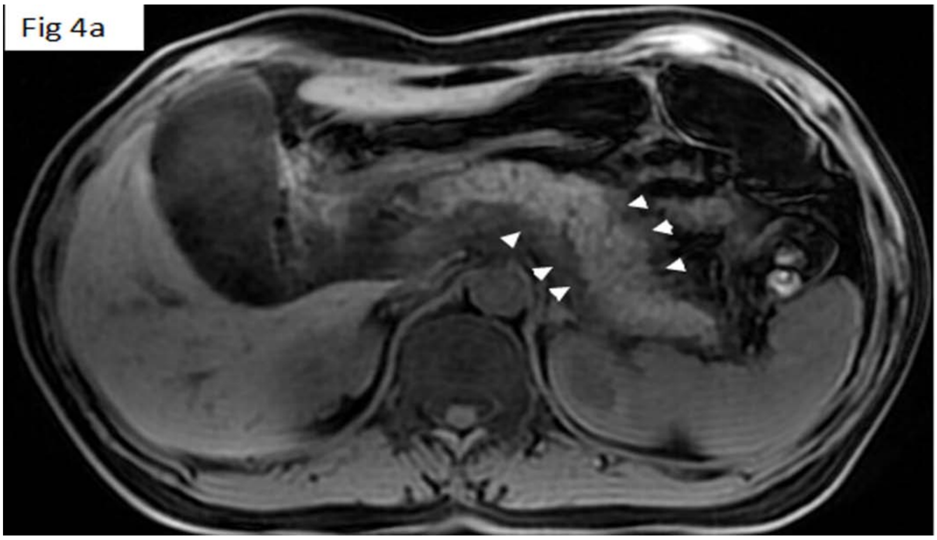

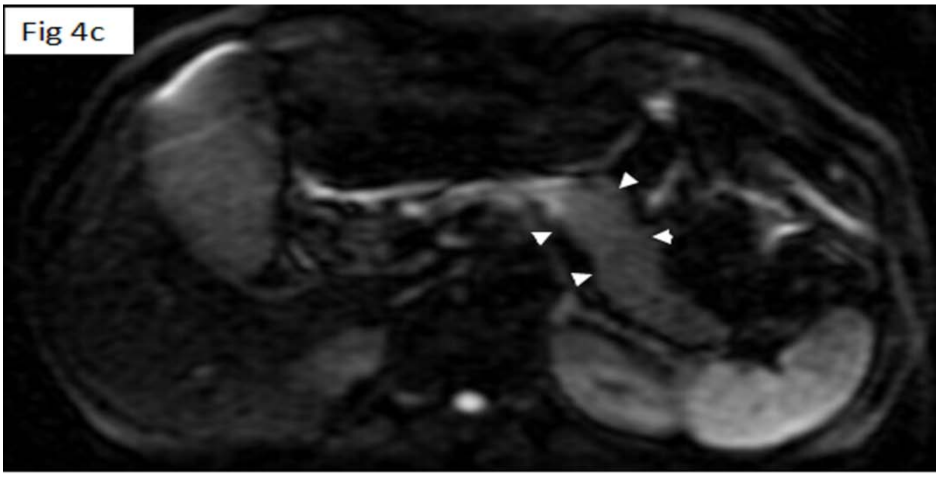

Supplement: S4 Fig — (PDF) [file pone.0160115.s006.pdf]
