# Supplementary material for: Preliminary Study of MR Diffusion Tensor Imaging of Pancreas for the Diagnosis of Acute Pancreatitis
Source: PLoS One. 2016 Sep 1;11(9):e0160115. doi: 10.1371/journal.pone.0160115 (PMC5008639; doi:10.1371/journal.pone.0160115)
Supplement: S5 Fig — (PDF) [file pone.0160115.s007.pdf]

S5 Fig: A 76-year-old woman with severe necrosis AP. Lava-flex T1 (a), FRFSE T2 (b), contrast Lava-flex T1(c), DTI(d) , ADC map (e) and FA map (f) weighted images show a well-marginated necrosis area without enhancement located in the whole pancreatic tissue(arrow) and a pseudocyst in omental sac.

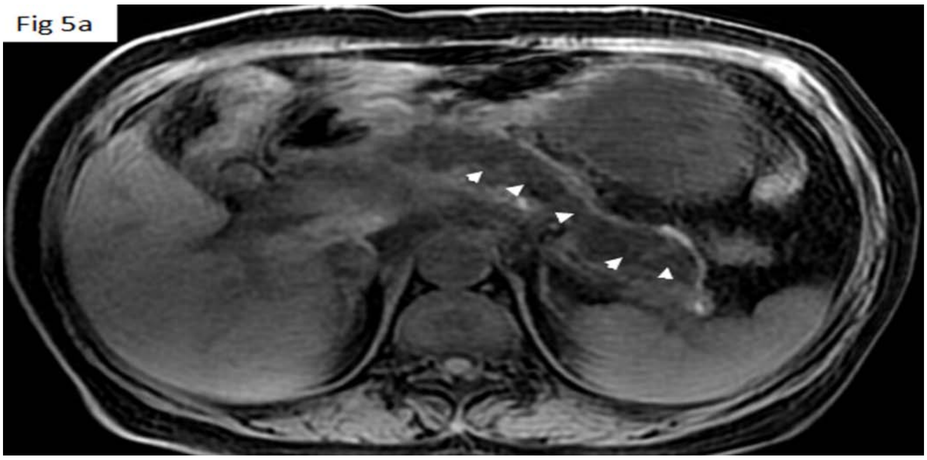

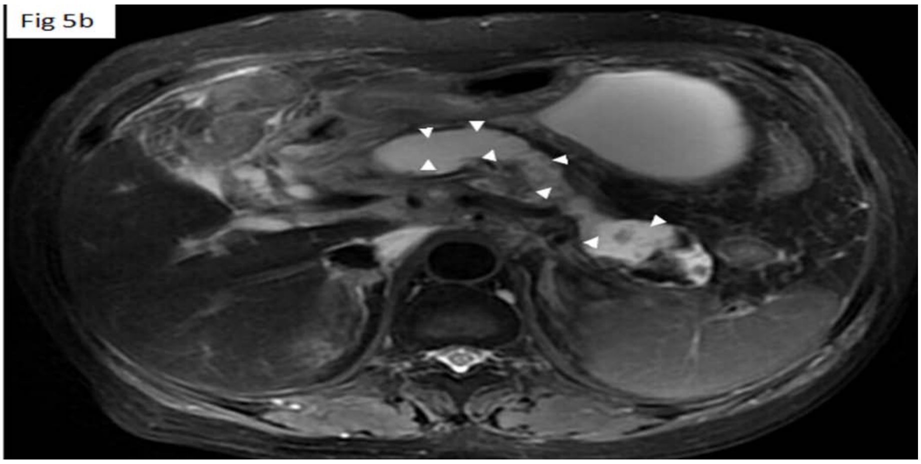

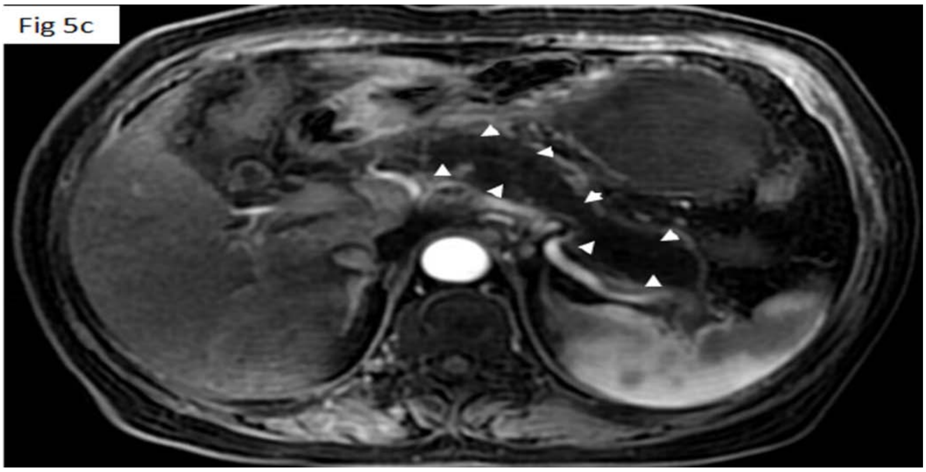

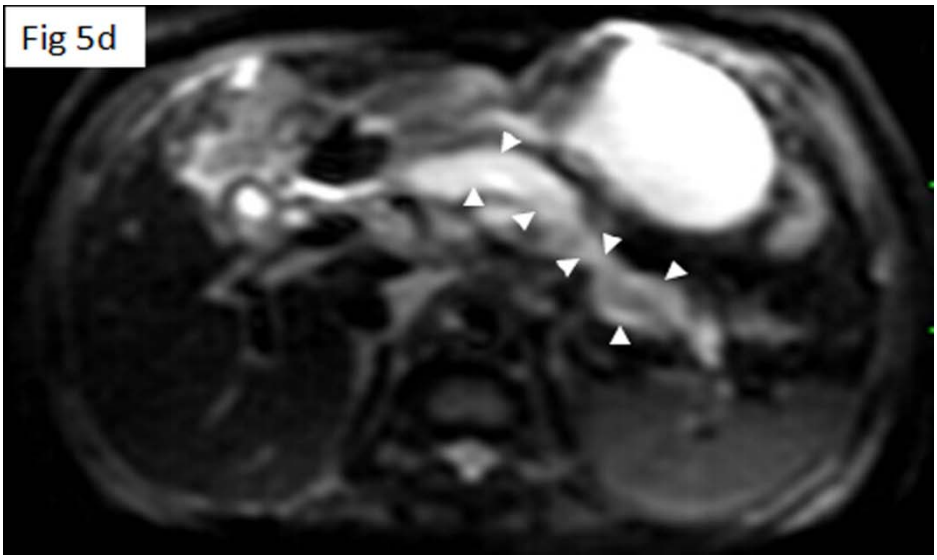

Fig 5d

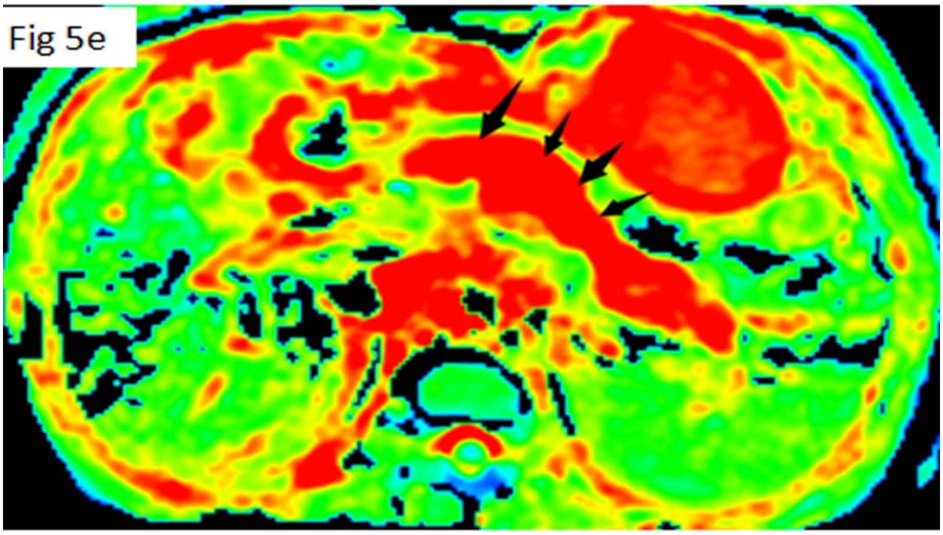

Fig 5e

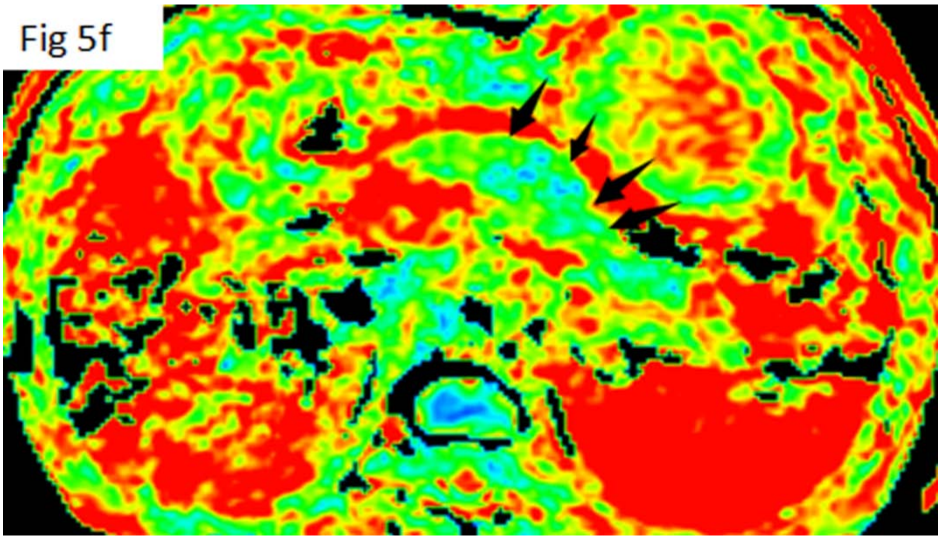

Fig 5f
