# Supplementary material for: Preliminary Study of MR Diffusion Tensor Imaging of Pancreas for the Diagnosis of Acute Pancreatitis
Source: PLoS One. 2016 Sep 1;11(9):e0160115. doi: 10.1371/journal.pone.0160115 (PMC5008639; doi:10.1371/journal.pone.0160115)

S6 Fig: The pancreatic mean ADC value (a) and FA value (b) contrast between in the AP group and normal group, we can gain that the pancreatic ADC value and FA value in the AP group was significantly lower than in the normal group.

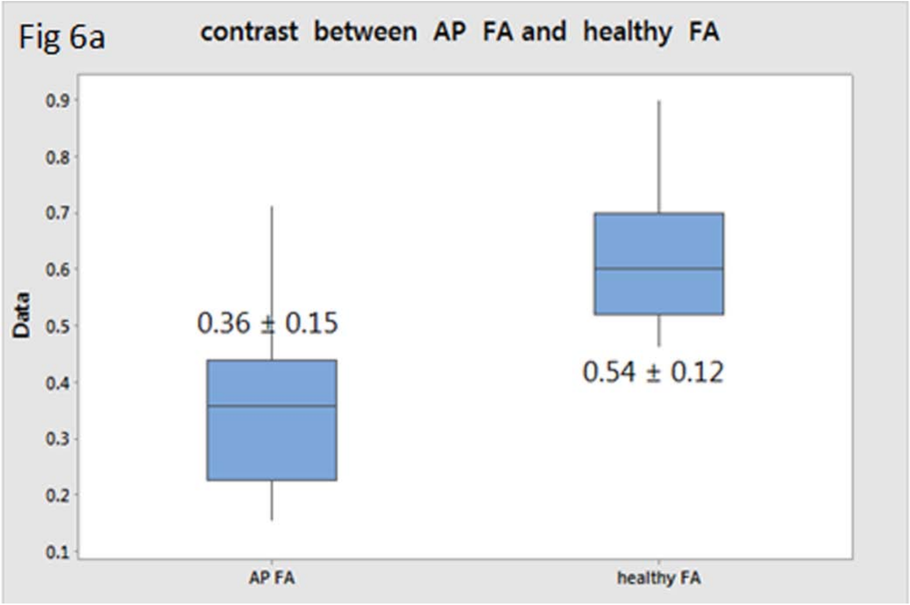

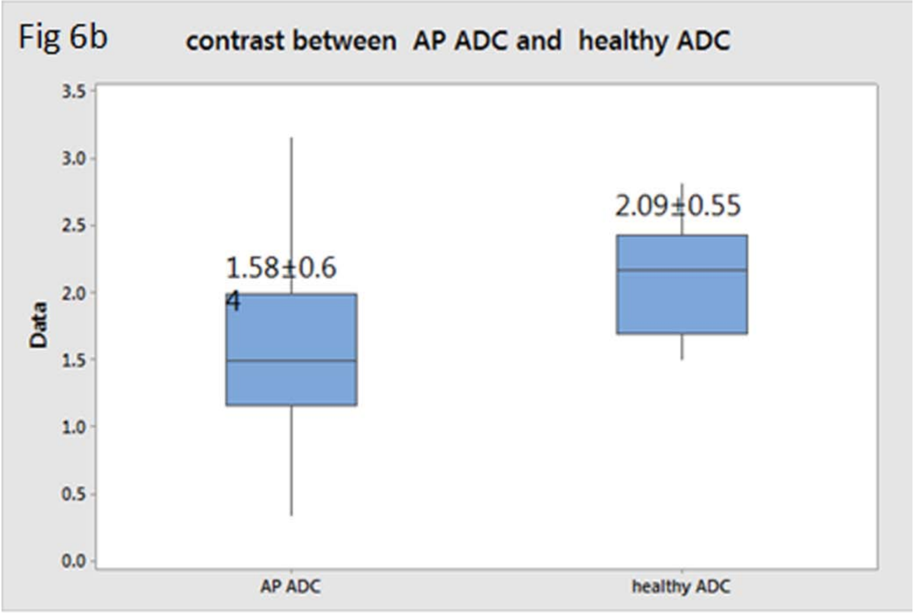

Supplement: S6 Fig — (PDF) [file pone.0160115.s008.pdf]
