# Supplementary material for: Preliminary Study of MR Diffusion Tensor Imaging of Pancreas for the Diagnosis of Acute Pancreatitis
Source: PLoS One. 2016 Sep 1;11(9):e0160115. doi: 10.1371/journal.pone.0160115 (PMC5008639; doi:10.1371/journal.pone.0160115)
Supplement: S1 Table — (PDF) [file pone.0160115.s009.pdf]

Table1. The parameters of routine pancreatic MR sequences at 3.0T.

	TR (ms)	TE (ms)	Flip angle	section thickness (mm)	intersection gap (mm)	matrix	FOV (cm)
AX 3D LAVA-Flex	4.2	2.6/1.3	15-20°	5	0	384×224	26–33
AX FRFSE T2WI	10000-12000	90–100	90°	5	0.5	256×192	36×34
COR SSFSE T2WI	2500-3500	80-100	90°	5	0.5	384×256	39×33
AX SSFSE T2WI	2500-3500	80-100	90°	5	0.5	320×256	39×33
MRCP	3045	1300	90°	40	40-50	384×224	32×34
AX 3D LAVA C+*	4.2	2.6/1.3	15-20°	5	0	384×224	26–33

Note: Dynamic enhanced imaging is indicated with*.Gadolinium chelate (Magnevist, Schering Guangzhou Co, China) was administered intravenously (0.2 mmol/L per kilogram of body weight) at approximately 3.5 mL/s using a double tube high-pressure injector (Spectris MR Injection System, Medrad Inc, USA) and was followed by a 20 mL saline solution flushed at the same speed. Affter the beginning of the injection, two arterial phase images were created in 19 seconds; two portal vein phase images in 60 seconds and one equilibrium phase image in 180 seconds were obtained.
