# Supplementary material for: Preliminary Study of MR Diffusion Tensor Imaging of Pancreas for the Diagnosis of Acute Pancreatitis
Source: PLoS One. 2016 Sep 1;11(9):e0160115. doi: 10.1371/journal.pone.0160115 (PMC5008639; doi:10.1371/journal.pone.0160115)
Supplement: S2 Table — (PDF) [file pone.0160115.s010.pdf]

**Table 2. MRSI scoring system.**

| Prognostic indicators | Characteristics | Points |
|---|---|---|
| Pancreatic inflammation | Normal pancreas | 0 |
| | Focal or diffuse enlargement of the pancreas | 1 |
| | Intrinsic pancreatic abnormalities with Inflammatory changes in the peripancreatic fat | 2 |
| | Single, poorly defined fluid collection or phlegmon | 3 |
| | Two or more poorly defined collection or presence of gas in or adjacent to the pancreas | 4 |
| Pancreatic necrosis | No necrosis | 0 |
| | <30% | 2 |
| | 30–50% | 4 |
| | >50% | 6 |
