# Supplementary material for: Preliminary Study of MR Diffusion Tensor Imaging of Pancreas for the Diagnosis of Acute Pancreatitis
Source: PLoS One. 2016 Sep 1;11(9):e0160115. doi: 10.1371/journal.pone.0160115 (PMC5008639; doi:10.1371/journal.pone.0160115)
Supplement: S3 Table — (PDF) [file pone.0160115.s011.pdf]

Table 3. Comparison of the FA and ADC value between edematous and necrotic AP.

Parameter	Edematous AP	Necrotic AP	*P* Value
ADC value	1.95 (0.44)	1.39 (0.18)	**0.000***
FA value	0.55(0.17)	0.18 (0.06)	**0.001***

Note: The data are the mean ADC and FA ((standard deviation). The ADC values were equal to mean value $\times 10^{-3}$ mm^{2}/s. Significant differences (P<0.05) are indicated with *.
