# Supplementary material for: Preliminary Study of MR Diffusion Tensor Imaging of Pancreas for the Diagnosis of Acute Pancreatitis
Source: PLoS One. 2016 Sep 1;11(9):e0160115. doi: 10.1371/journal.pone.0160115 (PMC5008639; doi:10.1371/journal.pone.0160115)
Supplement: S4 Table — (PDF) [file pone.0160115.s012.pdf]

Table 4 Correlation of the pancreas ADC and FA value with AP severity determined by the MRSI

ADC value ($\times 10^{-3} mm^2/s$)	AP subgroups based on MRSI			*R* value	*P* Value
	Mild (n=24)	Moderate (n=33)	Severe (n=9)		
ADC value	2.03 (0.52)	1.78 (0.19)	1.34(0.15)	**-0.635**	**0.003***
FA value	0.54(0.20)	0.49 (0.19)	0.16(0.04)	**-0.654**	**0.002***

Note: The data are the mean ADC and FA ((standard deviation). Significant differences ($P<0.05$) are indicated with *.
